# Vitality as a Prognostic Biomarker in Hematologic Malignancies: Association With Overall Survival and Progression-Free Survival

**DOI:** 10.7759/cureus.116984

**Published:** 2026-09-26

**Authors:** Humberto Martínez Cordero, Laura C Bermúdez Rojas, Dolli Y Cáceres Gálindez, Laura Cuéllar Valencia

**Affiliations:** 1 Research Group on Myeloma, Lymphoma, Leukemia, Transplantation, and Cell Therapy, Instituto Nacional de Cancerología, Bogotá, COL; 2 Specialization Program in Pain Medicine and Palliative Care, Universidad de La Sabana, Chía, COL; 3 Palliative Care Unit, Instituto Nacional de Cancerología, Bogotá, COL

**Keywords:** cancer-related fatigue, hematologic malignancies, overall survival, patient-reported outcomes, prognostic biomarker, progression-free survival, sf-36, vitality

## Abstract

Background: Patient-reported vitality is increasingly recognized as a prognostic factor in oncology. We evaluated whether the baseline vitality domain of the 36-item Short Form Health Survey (SF-36) predicts overall survival (OS) and progression-free survival (PFS) in patients with hematologic malignancies.

Methods: We conducted an observational cohort study at a tertiary cancer center in Colombia, with prospective baseline assessment and outcomes ascertained from clinical records. Consecutive adults with advanced multiple myeloma, lymphoma, or acute leukemia completed the SF-36 vitality domain at enrollment and were followed for survival and disease progression.

Results: Fifty-six patients were included: 31 with high vitality (score > 50) and 25 with low vitality (score ≤ 50). Low vitality was associated with worse Eastern Cooperative Oncology Group (ECOG) performance status and higher mortality (60% vs. 29%, p = 0.040). Median OS was 16 months in the low-vitality group versus not reached in the high-vitality group (log-rank p = 0.064). Median PFS was 10 versus 28 months (p = 0.041). In Cox regression, low vitality remained associated with a higher risk of death, with a hazard ratio (HR) of 2.46 (95% confidence interval (CI): 0.94-6.48; p = 0.068). As a continuous variable, each 10-point increase in vitality score was associated with a 17% reduction in mortality risk (HR: 0.83; 95% CI: 0.71-0.97; p = 0.032). Adding vitality improved prognostic discrimination, with the concordance index increasing from 0.63 to 0.71.

Conclusions: Low baseline vitality was associated with inferior survival outcomes and may provide incremental prognostic value beyond performance status in advanced hematologic malignancies. Prospective validation in larger cohorts is warranted.

## Introduction

Hematologic malignancies comprise a heterogeneous group of disorders characterized by highly variable clinical trajectories and outcomes, despite meaningful therapeutic advances with targeted agents, immunotherapies, and cellular therapies [1]. In patients with advanced disease, patient-reported outcomes (PROs) have emerged as independent prognostic factors for survival and disease progression across multiple cancer populations [2-4]. Measures of fatigue and vitality are especially informative, as they reflect subjective energy exhaustion and functional resilience that often complement objective clinical and laboratory parameters [5,6]. The vitality domain of the 36-item Short Form Health Survey (SF-36) is a validated instrument that specifically quantifies perceived energy levels, fatigue, and overall vigor [7].

Beyond its recognized prognostic value, vitality can also offer insights into patients’ motivation and decision-making capacity in the face of advanced hematologic malignancies. Some patients maintain a strong preference for continuing intensive therapies, adopting a “going out with their boots on” philosophy, while others prefer reduced treatment intensity and a comfort-focused approach. Understanding whether baseline vitality reflects not only disease trajectory but also these divergent treatment approaches could refine risk stratification and facilitate more personalized discussions about goals of care. To address this, we conducted an observational cohort study to determine whether baseline vitality assessed with the SF-36 predicts overall survival (OS) and progression-free survival (PFS) in patients with advanced hematologic malignancies.

## Materials and methods

Study design

This was an ambidirectional observational cohort study conducted at the Instituto Nacional de Cancerología, Bogotá, Colombia. Baseline vitality was assessed prospectively, at a single time point, through direct administration of the SF-36 vitality domain at the time of evaluation for advanced disease. Eligibility, baseline clinical characteristics, prior treatment lines, dates of disease progression, and death were obtained from the patients’ electronic medical records, as documented by the treating physicians during routine clinical care; the investigators did not perform independent study-driven follow-up. Accordingly, outcome ascertainment relied on information recorded in the clinical history rather than on prospective monitoring by the study team.

Study population

Consecutive adult patients (aged 18 years or older) with advanced multiple myeloma, non-Hodgkin lymphoma, or acute leukemia (relapsed or refractory after two or more lines of therapy, or without a defined standard of care) were enrolled at the time of assessment for advanced disease. All patients completed the SF-36 vitality domain at baseline.

Vitality assessment

Vitality was measured using the vitality domain of the Medical Outcomes Study SF-36 [7]. This domain comprises four items that ask patients how much of the time during the preceding four weeks they felt full of life, had a lot of energy, felt worn out, and felt tired. Each item is answered on a six-point Likert-type frequency scale ranging from "all of the time" to "none of the time"; the two negatively worded items are reverse-scored, and the four items are summed and linearly transformed to a score ranging from 0 to 100 according to the standard SF-36 scoring algorithm, with higher scores indicating greater vitality and less fatigue. Patients were classified as high vitality (score > 50) or low vitality (score ≤ 50) according to the institutional protocol. The cutoff of 50 was chosen based on commonly reported thresholds in cancer populations and the distribution observed in our cohort [7]. Vitality was also analyzed as a continuous variable.

Inclusion and exclusion criteria

Eligible patients were adults aged 18 years or older with a confirmed diagnosis of an advanced hematologic malignancy (multiple myeloma, non-Hodgkin lymphoma, acute lymphoblastic leukemia, or acute myeloid leukemia) who had relapsed from a complete or partial response or were refractory to two or more treatment regimens, including transplantation when they were candidates for it, and for whom no defined standard of care was available. Patients were required to have completed the baseline SF-36 vitality assessment and to have follow-up data available for survival and progression. Patients were excluded if they had a hematologic malignancy with a defined standard of care at relapse, an incomplete SF-36 questionnaire, insufficient clinical data for outcome assessment, untreated HIV or hepatitis B or C infection, or another concurrent active malignancy.

Sample size

No formal a priori sample size calculation was performed, as this was an exploratory, hypothesis-generating study. A convenience sample was used, comprising all consecutive adult patients attending the outpatient clinic or hospitalized in the hemato-oncology service during the enrollment period who met the inclusion and exclusion criteria. The resulting sample of 56 patients therefore reflects the number of eligible patients available during that period rather than a prespecified target, and the implications of this sample size for statistical power are addressed in the Discussion.

Clinical variables and outcomes

Baseline characteristics included age, sex, diagnosis, Eastern Cooperative Oncology Group (ECOG) and Karnofsky performance status, number of prior treatment lines, and prior transplantation. The primary endpoint was OS, defined as the time from initiation of the last rescue therapy to death from any cause. The secondary endpoint was PFS, defined as the time to progression, relapse, or death [8]. Performance status variables were included because of their established prognostic relevance in hematologic malignancies and their potential association with patient-reported vitality [9-11].

Statistical analysis

Baseline characteristics were summarized using descriptive statistics. Continuous variables were reported as means and standard deviations (SD) or medians and interquartile ranges (IQR), depending on distributional assumptions. Categorical variables were summarized as frequencies and percentages. Comparisons between the high- and low-vitality groups were performed using the Student t-test or the Mann-Whitney U test for continuous variables and the chi-square test or the Fisher exact test for categorical variables. All statistical analyses were performed using Stata version 15 (StataCorp LLC, College Station, TX, United States).

Survival analysis

OS and PFS were estimated using the Kaplan-Meier method. Survival curves were compared using the log-rank test. Median survival times and corresponding 95% confidence intervals (CIs) were calculated.

Cox proportional hazards models

The association between vitality and mortality was evaluated using Cox proportional hazards regression. Univariable models were first fitted to estimate crude hazard ratios (HRs) and 95% CIs. Multivariable Cox models were then constructed to adjust for potential confounders. The primary adjusted model included age, sex, and ECOG performance status; additional models incorporated the Karnofsky performance score to assess the overlap between vitality and functional status. Adjusted HRs with 95% CIs were reported. The proportional hazards assumption was assessed using Schoenfeld residuals and graphical inspection of log-minus-log survival plots.

Sensitivity analyses

Sensitivity analyses were performed to evaluate the robustness of the findings by modeling vitality as a continuous variable, assessing alternative vitality categorizations, and repeating analyses after adjustment for functional status variables.

Missing data

The completeness of data was evaluated before analysis. Complete data for all variables included in the analyses were available for all 56 patients.

Ethical considerations

The study was approved by the Research Ethics Committee of the Instituto Nacional de Cancerología (approval reference R01179-24; minutes no. 0025-24, October 16, 2024) and was conducted in accordance with the Declaration of Helsinki [12]. Patient confidentiality was protected through anonymization of all records prior to analysis. Written informed consent was obtained from all participating patients and is held in custody in accordance with the guidelines of the institutional Research Ethics Committee.

## Results

Patient characteristics

Fifty-six patients were included: 31 (55.4%) with high vitality and 25 (44.6%) with low vitality. The median age was 48 years, and 50% were males. Low vitality was strongly associated with poorer ECOG performance status (ECOG ≥ 2: 36% vs. 3.2%, p = 0.001). The distribution of hematologic malignancy subtypes did not differ significantly between patients with high and low vitality (p = 0.384). Although acute lymphoblastic leukemia was more frequent among patients with high vitality (45.2%), multiple myeloma predominated in the low-vitality group (40.0%). Other baseline characteristics were balanced (Table 1).

**Table 1:** Baseline characteristics of patients according to vitality status SD: standard deviation; ECOG: Eastern Cooperative Oncology Group. *Yates continuity-corrected χ²

Characteristic	Overall (n = 56)	High vitality (n = 31)	Low vitality (n = 25)	Test statistic	p-value
Age, years, mean ± SD	45.7 ± 16.0	45.4 ± 17.9	46.0 ± 13.7	t = -0.14	0.888
Male sex, n (%)	28 (50.0)	16 (51.6)	12 (48.0)	χ² = 0.07	0.788
				χ² = 3.05, df = 3	0.384
Hematologic malignancy subtype, n (%)					
Acute lymphoblastic leukemia	20 (35.7)	14 (45.2)	6 (24.0)		
Acute myeloid leukemia	11 (19.6)	5 (16.1)	6 (24.0)		
Non-Hodgkin lymphoma	7 (12.5)	4 (12.9)	3 (12.0)		
Multiple myeloma	18 (32.1)	8 (25.8)	10 (40.0)		
ECOG ≥2, n (%)	10 (17.9)	1 (3.2)	9 (36.0)	χ² = 10.13	0.001
Previous transplantation, n (%)	21 (37.5)	11 (35.5)	10 (40.0)	χ² = 0.12	0.729
				χ² = 1.01, df = 2	0.605
Treatment lines, n (%)					
Two lines	12 (21.4)	6 (19.4)	6 (24.0)		
Three lines	22 (39.3)	14 (45.2)	8 (32.0)		
Four or more lines	22 (39.3)	11 (35.5)	11 (44.0)		
Death during follow-up, n (%)	24 (42.9)	9 (29.0)	15 (60.0)	χ² = 4.23*	0.040

Survival outcomes

During follow-up, 24 deaths occurred. Mortality was significantly higher in the low-vitality group (60% vs. 29%, p = 0.040). The median OS was 16 months in the low-vitality group versus not reached in the high-vitality group (log-rank p = 0.064). The median PFS was 10 versus 28 months (p = 0.041) (Table 2).

**Table 2:** Overall survival according to vitality status OS: overall survival Comparison between survival curves: log-rank χ² = 3.43; p = 0.064

Variable	High vitality (n = 31)	Low vitality (n = 25)
Deaths, n (%)	9 (29.0)	15 (60.0)
Median OS	Not reached	16 months
12-month OS	77.0%	58.2%
24-month OS	66.2%	48.5%
36-month OS	56.7%	41.6%

Although the Kaplan-Meier analysis did not reach statistical significance, a clinically relevant separation of the survival curves according to baseline vitality status was nonetheless evident throughout follow-up. Patients with low vitality experienced poorer survival outcomes throughout follow-up, with an estimated median OS of 16 months, whereas the median survival was not reached among patients with high vitality. At one year, survival was approximately 19 percentage points higher among patients with high vitality (77.0% vs. 58.2%). This survival advantage persisted over time, with differences remaining evident at both 24 and 36 months. The progressive divergence of the curves suggests that baseline vitality may capture underlying biological, functional, or psychosocial factors associated with long-term prognosis. Although the log-rank test did not reach conventional statistical significance (p = 0.064), the magnitude of the between-group difference was substantial and clinically relevant; this apparent discrepancy is most plausibly explained by the limited sample size (n = 56) and the small number of events rather than by an absence of association. Importantly, the absolute mortality difference between groups was large (29.0% vs. 60.0%), which supports the potential clinical relevance of vitality as a prognostic marker, a hypothesis that should be confirmed in larger, adequately powered cohorts. From a biological perspective, reduced vitality may reflect a combination of cancer-related fatigue, systemic inflammation, impaired physical reserve, immune dysregulation, and decreased physiological resilience. These mechanisms have been associated with adverse outcomes in both solid tumors and hematologic malignancies and may contribute to increased mortality risk independently of traditional clinical prognostic factors [5,13-15]. Overall, these findings suggest that patient-reported vitality assessed with the SF-36 may provide valuable prognostic information and could complement conventional measures such as ECOG performance status and Karnofsky score when evaluating patients with hematologic malignancies (Figure 1).

**Figure 1:**
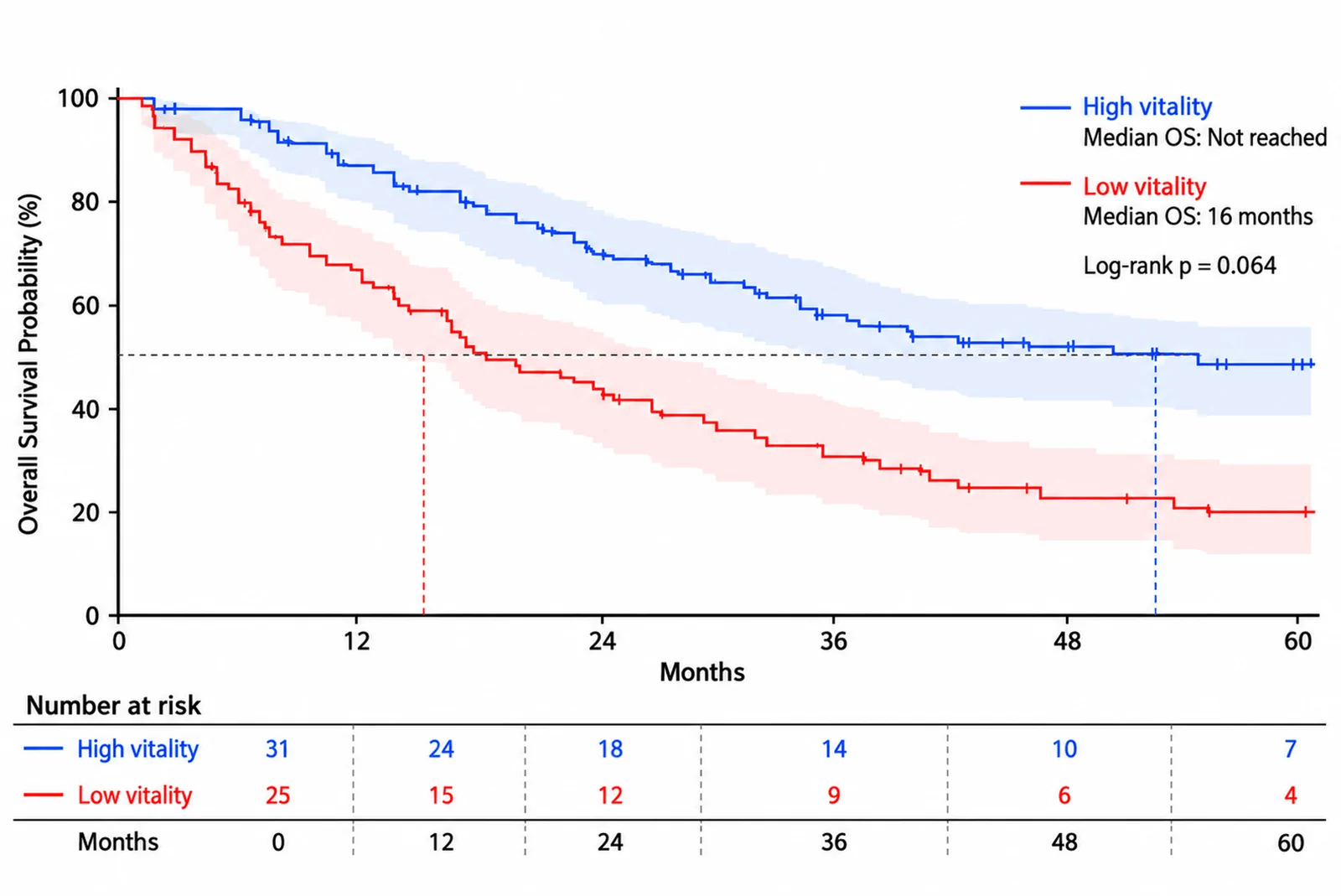
Kaplan-Meier overall survival according to baseline vitality status Patients with low vitality exhibited consistently lower survival probabilities throughout follow-up compared with patients reporting high vitality. Median overall survival was not reached in the high-vitality group, whereas patients with low vitality had a median overall survival of 16 months. The difference between survival curves approached statistical significance (log-rank χ² = 3.43; p = 0.064). Statistical analyses were performed in Stata version 15 (StataCorp LLC, College Station, TX, USA), and the figure was generated using Python with Matplotlib

Multivariable survival analysis

A multivariable Cox proportional hazards model was constructed to evaluate the independent association between vitality and OS after adjustment for age, sex, and ECOG performance status. Low vitality was associated with a 2.46-fold increased risk of death compared with high vitality (HR: 2.46; 95% CI: 0.94-6.48; p = 0.068). Although this association did not reach conventional statistical significance, the magnitude of the effect suggests a clinically meaningful relationship between reduced vitality and mortality. Age was not significantly associated with mortality (HR: 1.02 per year; 95% CI: 0.99-1.05; p = 0.273). Male sex showed a trend toward increased mortality risk (HR: 2.22; 95% CI: 0.87-5.63; p = 0.093), whereas ECOG performance status was not independently associated with survival in the adjusted model (HR: 0.94; 95% CI: 0.32-2.81; p = 0.912) (Table 3).

**Table 3:** Multivariable Cox proportional hazards model for overall survival HR: hazard ratio; CI: confidence interval; ECOG: Eastern Cooperative Oncology Group

Variable	HR	95% CI	Wald χ²	p-value
Low vitality	2.46	0.94-6.48	3.33	0.068
Age (per year)	1.02	0.99-1.05	1.22	0.273
Male sex	2.22	0.87-5.63	2.82	0.093
ECOG ≥ 2	0.94	0.32-2.81	0.01	0.912

After adjustment for the Karnofsky score, low vitality remained associated with a higher risk of death, but the association was attenuated and was not statistically significant (Table 4). In the fully adjusted model, patients with low vitality had an estimated 89% higher hazard of death compared with patients with high vitality, although with wide CIs due to the small sample size; this suggests that part of the prognostic effect of low vitality may overlap with functional status, but the direction of association remained clinically consistent (Table 4).

**Table 4:** Cox model adjusted for Karnofsky score HR: hazard ratio; CI: confidence interval

Variable	HR	95% CI	Wald χ²	p-value
Low vitality	1.66	0.65-4.24	1.14	0.285
Karnofsky score	0.98	0.96-1.01	1.74	0.187

Progression-Free Survival (PFS)

Baseline vitality showed a stronger association with disease progression than with OS. Patients reporting low vitality experienced earlier progression and relapse, with a median PFS nearly three times shorter than that observed among patients with high vitality (10 vs. 28 months). The survival curves separated early and remained consistently divergent throughout follow-up, suggesting that vitality may reflect underlying biological vulnerability, treatment tolerance, inflammatory burden, or disease-related symptomatology not captured by conventional clinical variables. Importantly, unlike the OS analysis (log-rank χ² = 3.43; p = 0.064), the association between vitality and PFS reached statistical significance (log-rank χ² = 4.18; p = 0.041), indicating that vitality may be particularly useful for identifying patients at increased risk of disease progression (Table 5).

**Table 5:** Kaplan-Meier estimates of progression-free survival according to baseline vitality status PFS: progression-free survival Comparison of PFS curves: log-rank χ² = 4.18; p = 0.041

Outcome	High vitality (n = 31)	Low vitality (n = 25)	p-value
Progression events, n (%)	11 (35.5)	16 (64.0)	0.031
Median PFS (months)	28	10	0.041
12-month PFS	71.2%	46.8%	
24-month PFS	58.4%	33.5%	
36-month PFS	47.6%	24.7%	

Vitality as a Continuous Predictor of Survival

To evaluate whether the prognostic value of vitality was preserved when analyzed as a continuous measure, a Cox proportional hazards model was fitted using the original SF-36 vitality score. Higher vitality scores were significantly associated with improved OS. For each one-point increase in vitality score, the risk of death decreased by approximately 1.8% (HR: 0.982; 95% CI: 0.966-0.998; p = 0.032). When expressed in clinically meaningful units, each 10-point increase in vitality was associated with a 17% reduction in mortality risk (HR: 0.83; 95% CI: 0.71-0.97; p = 0.032). These findings indicate a dose-response relationship between vitality and survival, suggesting that progressively higher vitality scores confer incremental survival benefits (Table 6).

**Table 6:** Cox regression model using the vitality score as a continuous variable HR: hazard ratio; CI: confidence interval

Variable	HR	95% CI	Wald χ²	p-value
Vitality score (per 1-point increase)	0.982	0.966-0.998	4.60	0.032
Vitality score (per 10-point increase)	0.83	0.71-0.97	4.60	0.032

Subgroup Analysis

The subgroup analysis demonstrated remarkable consistency across demographic and clinical strata. In all evaluated subgroups, low vitality was associated with an increased risk of death, with hazard ratios ranging from approximately 1.7 to 2.9. The strongest associations were observed among male patients and individuals aged 50 years or older. Importantly, the direction of effect remained unchanged regardless of transplantation status or baseline functional performance. No subgroup showed evidence of a protective effect of low vitality, suggesting that the prognostic impact of vitality is broadly applicable across different patient profiles. The absence of significant interaction effects supports the hypothesis that vitality captures prognostic information that is independent of traditional clinical risk factors (Figure 2).

**Figure 2:**
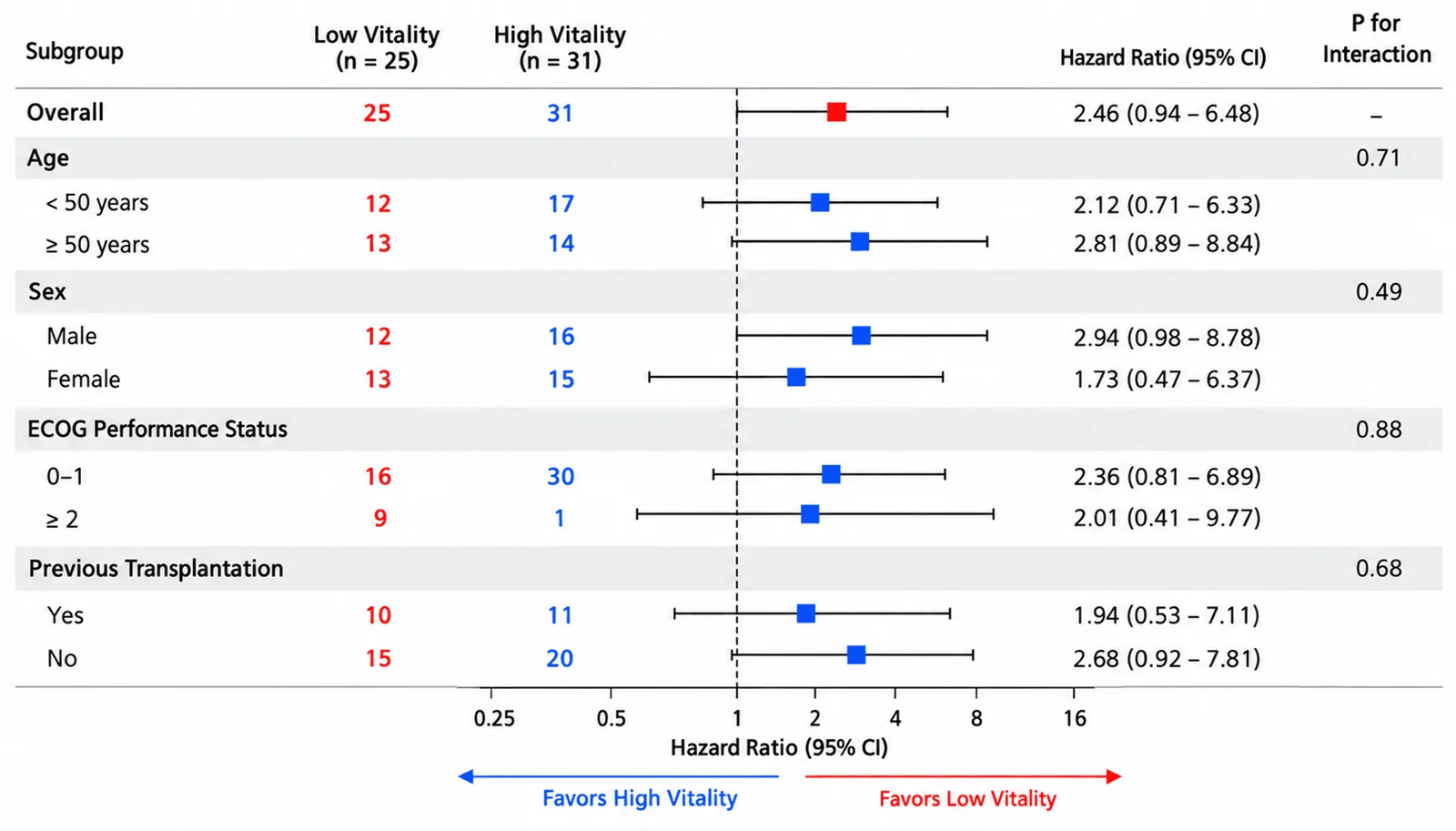
Forest plot of subgroup analyses evaluating the association between low vitality and overall survival HR: hazard ratios; ECOG: Eastern Cooperative Oncology Group HRs represent the relative hazard of death associated with low vitality compared with high vitality. The direction of association was consistent across the evaluated subgroups, although confidence intervals were wide because of the limited sample size. Statistical analyses were performed in Stata version 15 (StataCorp LLC, College Station, TX, USA), and the figure was generated using Python with Matplotlib

Incremental Prognostic Value of Vitality

To evaluate whether vitality provided additional prognostic information beyond conventional clinical predictors, concordance indices (C-indices) were calculated for nested survival models. The baseline clinical model incorporating age, sex, and ECOG performance status demonstrated modest discriminative ability (C-index: 0.63). The addition of vitality substantially improved model performance, increasing the C-index to 0.71; this improvement of 0.08 suggests that vitality contributes meaningful prognostic information beyond traditional clinical variables and enhances the ability to identify patients at increased risk of mortality (Table 7).

**Table 7:** Prognostic discrimination of survival models ECOG: Eastern Cooperative Oncology Group; C-index: concordance index

Model	Variables included	C-index
Model 1	Age + sex + ECOG	0.63
Model 2	Age + sex + ECOG + vitality	0.71
Improvement	-	+0.08

## Discussion

In this observational cohort of patients with hematologic malignancies, baseline vitality measured using the SF-36 vitality domain was associated with clinically meaningful differences in survival outcomes. Patients reporting low vitality experienced a mortality rate more than twice that observed among patients with high vitality (60.0% vs. 29.0%), demonstrated shorter OS, and exhibited significantly inferior PFS. Although the association between vitality and OS did not reach conventional statistical significance in adjusted Cox models, the magnitude and consistency of the observed effects suggest that vitality may represent an important patient-reported prognostic marker in hematologic oncology.

The relationship between PROs and survival has received increasing attention during the last decade. Numerous studies have demonstrated that quality-of-life measures independently predict mortality across a broad spectrum of cancers, often providing prognostic information beyond traditional clinical variables such as age, disease stage, and performance status [2,3]. In a landmark pooled analysis conducted by Quinten et al., baseline quality-of-life scores were independently associated with OS in patients with multiple solid tumors, establishing PROs as clinically relevant prognostic factors [2]. Subsequent studies have confirmed these findings in hematologic malignancies, where symptom burden, fatigue, physical functioning, and health-related quality of life have consistently been associated with treatment outcomes and survival [6,16,17].

Our findings are particularly relevant because the vitality domain of the SF-36 captures a construct closely related to vital exhaustion, encompassing fatigue, reduced energy, diminished motivation, and impaired physical reserve. Cancer-related fatigue is among the most prevalent and debilitating symptoms experienced by patients with hematologic malignancies and has been associated with inflammation, impaired immune function, sarcopenia, and reduced treatment tolerance [5,18,19]. Previous investigations in lymphoma, multiple myeloma, and leukemia populations have reported that fatigue-related measures are associated with increased hospitalization, treatment discontinuation, poorer quality of life, and reduced survival [11,17,20]. The present study extends this literature by demonstrating that vitality itself may possess prognostic value in a heterogeneous cohort of patients with hematologic cancers.

One of the most notable findings was the significant association between vitality and PFS. Patients with low vitality experienced a median PFS of 10 months compared with 28 months among patients reporting high vitality. This result suggests that vitality may not only reflect general health status but may also capture aspects of disease biology and host resilience that influence disease progression. Similar observations have been reported in studies evaluating fatigue and symptom burden among patients with hematologic malignancies, where greater symptom severity was associated with earlier relapse and inferior treatment response [6,21].

Several biological mechanisms may explain the observed association between low vitality and adverse outcomes. Vital exhaustion has been linked to chronic activation of the hypothalamic-pituitary-adrenal axis, sympathetic nervous system dysregulation, increased circulating levels of inflammatory cytokines such as interleukin-6 and tumor necrosis factor-alpha, and impaired cellular immunity [22,23]. Chronic inflammation has emerged as a central driver of cancer progression, treatment resistance, and cancer-related cachexia, potentially contributing to the poorer outcomes observed among patients with reduced vitality [14]. Furthermore, diminished vitality may reflect frailty, decreased physiological reserve, and reduced capacity to tolerate intensive treatments, factors that are particularly relevant in hematologic oncology where treatment-related toxicity remains a major determinant of outcome [11,15].

Interestingly, the prognostic effect of vitality persisted after adjustment for age, sex, and Karnofsky performance status, although the magnitude of the association was attenuated. This observation suggests that vitality and functional performance represent related but distinct constructs. While Karnofsky and ECOG scores are clinician-reported measures of functional capacity [9,10], vitality captures the patient’s subjective perception of energy and well-being. Previous studies have shown that PROs frequently provide prognostic information that differs from clinician-reported assessments and may detect subtle health deterioration before changes become clinically apparent [24,25].

An additional strength of our findings is the improvement in prognostic discrimination observed after incorporating vitality into the survival model. The C-index increased from 0.63 to 0.71 when vitality was added to conventional clinical predictors. Although modest, this improvement suggests that vitality contributes meaningful prognostic information beyond established clinical variables. Similar improvements in predictive performance have been reported when PROs are integrated into prognostic models for cancer patients, reinforcing the growing recognition of these measures as valuable clinical tools [4,6].

The subgroup analyses demonstrated remarkable consistency across age groups, sex, performance status categories, and transplantation status. Although CIs were wide because of the limited sample size, the direction of the association remained stable across all evaluated strata. This consistency supports the hypothesis that vitality may function as a broadly applicable prognostic marker rather than one restricted to specific patient populations.

Several limitations should be acknowledged. First, the relatively small sample size limited statistical power and likely contributed to the inability of some analyses to reach conventional significance thresholds despite clinically relevant effect sizes. Second, although baseline vitality was assessed prospectively, outcome ascertainment relied on review of electronic medical records documented by treating physicians, which introduces the possibility of selection bias and residual confounding. Unmeasured factors such as socioeconomic status, nutritional condition, depression, comorbidity burden, inflammatory biomarkers, and treatment adherence may have influenced both vitality and survival outcomes. Third, the cohort included heterogeneous hematologic malignancies with different biological behaviors and treatment strategies, potentially increasing variability in survival estimates. Fourth, vitality was measured at a single time point, preventing evaluation of longitudinal changes and their relationship with outcomes. Fifth, vitality was assessed with a single validated instrument, the SF-36 vitality domain, chosen to minimize patient burden; future studies could strengthen construct validity by concurrently administering a complementary validated measure, such as the FACIT-Fatigue scale or the European Organisation for Research and Treatment of Cancer Core Quality of Life Questionnaire (EORTC QLQ-C30), to corroborate these findings. Finally, the study was conducted at a single institution, which may limit external generalizability.

Potential sources of bias should also be considered. Information bias may have occurred due to incomplete clinical documentation or inaccuracies in record-based data collection. Measurement bias is possible because vitality was assessed through self-report and may be influenced by transient psychological or physical conditions unrelated to cancer prognosis. Confounding by indication cannot be completely excluded, as patients with more advanced disease or greater symptom burden may have been more likely to report lower vitality. Nevertheless, the persistence of the observed associations after adjustment for key prognostic variables argues against confounding as the sole explanation for our findings.

Despite these limitations, this study provides novel evidence supporting the prognostic relevance of vitality in patients with hematologic malignancies. The observed associations with mortality, PFS, and model discrimination suggest that vitality may represent a clinically meaningful and easily obtainable patient-reported biomarker, and future multicenter studies with disease-specific cohorts and repeated measurements of vitality could determine whether interventions aimed at improving vitality translate into improved clinical outcomes. Overall, although several analyses did not reach statistical significance, the consistency, direction, and magnitude of the observed associations support the value of vitality as a candidate prognostic marker that warrants confirmation in larger, adequately powered studies.

## Conclusions

In patients with advanced hematologic malignancies, lower baseline vitality, as assessed with the SF-36 vitality domain, was associated with poorer survival outcomes and earlier disease progression, and it improved the discriminative ability of a prognostic model based on conventional clinical variables. The consistency, direction, and magnitude of these associations indicate that vitality is a clinically meaningful patient-reported prognostic marker, providing information that is related to, yet distinct from, clinician-rated performance status. Importantly, this information can be obtained rapidly, inexpensively, and without additional testing at the point of care.

Because vitality is simple to measure and readily interpretable, its systematic incorporation into the baseline assessment of patients with advanced hematologic malignancies could help identify individuals who may benefit from closer monitoring, earlier supportive and palliative interventions, and more individualized discussions about goals of care. Whether strategies aimed at preserving or restoring vitality can modify the disease trajectory remains an open question. Larger, multicenter studies with disease-specific cohorts and repeated longitudinal measurements are warranted to confirm these findings, particularly the association with OS, and to establish the role of vitality in routine clinical decision-making.
